# Gallium Oxide-Based Photodetectors for Water Quality Monitoring

**DOI:** 10.1021/acsaom.5c00620

**Published:** 2026-02-04

**Authors:** David Nicol, Aurora Uras, Nathalie Lidgi-Guigui, William J. Peveler, Núria Martínez-Carreras, Fabien C−P. Massabuau

**Affiliations:** † Department of Physics, 3527 SUPA, University of Strathclyde, Glasgow G4 0NG, U.K.; ‡ Laboratoire des Sciences des Procédés et des Matériaux (LSPM), CNRS, University of Sorbonne Paris-Nord, 93430 Villetaneuse, France; § School of Chemistry, 3526 University of Glasgow, Glasgow G12 8QQ, U.K.; ∥ Catchment and Eco-hydrology Research Group (CAT), Environmental Sensing and Modelling Unit (ENVISION), Luxembourg Institute of Science and Technology, L-4422 Belvaux, Luxembourg

**Keywords:** gallium oxide, photodetector, ultraviolet, water, nitrates, organic carbon

## Abstract

We present an approach to water quality monitoring using gallium oxide (Ga_2_O_3_) ultrawide-band-gap semiconductors. Nitrates, dissolved organic carbon, and suspended solid concentrations are three commonly measured water quality parameters that display optical absorption ranging from the deep ultraviolet to the visible region. This broad spectral region poses a challenge for accurate and efficient (simultaneous) measurement of absorption/extinction arising from varying concentrations of these parameters because silicon (Si), the classical detector material, has poor performance across this optical region. To overcome these limitations, we propose the use of ultrawide-band-gap semiconductors to trace changes in optical absorption from varying water compositions by measuring the photocurrent response at different wavelengths. Here, we use α-phase Ga_2_O_3_ as a suitable material to measure a broad photocurrent response ranging from 200 to 465 nm. The photocurrent response consisted of three well-defined regions inherently linked to the rich electronic landscape of the material. Region (i) (200–250 nm) corresponds to band-to-band excitation of charge carriers, aligning well with the absorption characteristics of nitrates. Region (ii) (250–350 nm) corresponds to band tail-related transitions, allowing a photocurrent response to dissolved organic carbon concentrations. Finally, we utilize defect-mediated transitions in Region (iii) (350–465 nm) to monitor suspended solid concentrations. It was observed here that the sensitivity of the photocurrent response to the changing water composition strongly depends on the excitation wavelength, where 225, 260, and 465 nm excitation yielded (for our setup) the best results for the monitoring of nitrates, dissolved organic carbon, and suspended solid concentrations, respectively.

## Graphical abstract

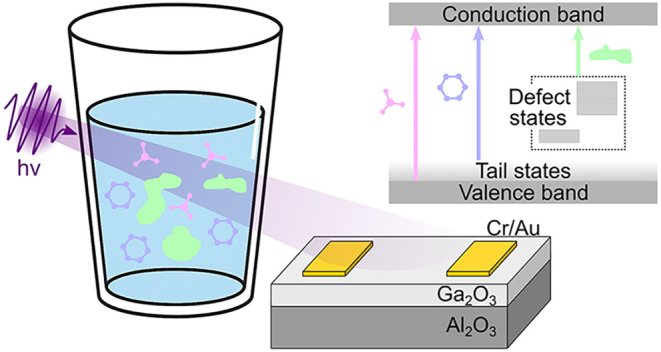

## Introduction

Ensuring access to clean water is of paramount human and ecological importance. It is estimated that around 1 billion people worldwide lack access to clean water, resulting in over 2 million deaths per year.^1^ With population rapidly expanding, and industrial applications reducing the availability of water which is safe for human consumption, there is a growing need to effectively monitor the quality of water.^2,3^ The importance of water quality has been recognized at an international level, with the United Nations listing clean water and sanitation as a Sustainable Development Goal, with the target of establishing clean water for all by 2030.^1^ The first step to reduce harmful contaminants in water sources is to develop effective methods to monitor their concentrations.

The concentration of certain compounds in water can serve as an excellent metric to define water quality. Three common impurities are nitrates, dissolved organic carbon (DOC), and suspended solid concentration (SSC). Nitrates are compounds that are formed in the natural nitrogen cycle and used in many applications ranging from fertilizers to explosives. Nitrates are the most common contaminants in water and can have a detrimental effect on human health by enhancing pathogenesis of some gastric cancers.^4,5^ Furthermore, elevated nitrate levels will have adverse effects on aquatic animals, causing histopathological alterations in the gills, esophagus, and brain.^6^ Nitrate incorporation in aquatic life will inevitably have repercussions on human consumption via the food chain. DOC can come from a variety of sources but usually from the decomposition of dead organic matter, including plants and wildlife. It can have staggering effects on water quality due to its ability to form complexes to alter the mobility of heavy metals and challenges the efficiency of water treatment processes.^7^ Finally, SSC is a measure of suspended solids residing in water. Suspended solids in large concentrations reduce light penetration and can act as an absorber altering the physical, chemical, and biological properties of water. Increased water temperatures and reduced dissolved oxygen levels through a reduction in the photosynthesis process are linked to high SSC levels absorbing light.^8^ To deliver the United Nations’ Sustainable Development Goal 6 of access to clean water for all, it will be critical to develop solutions to effectively monitor nitrate, DOC, and SSC levels in water in a convenient way that can be widespread, energy-efficient, and simple to use.

Recently UV–vis spectrophotometry has been gaining attention as an effective water monitoring method able to trace impurity concentrations over a wide spectral range.^9^ The aforementioned water quality parameters exhibit different absorption characteristics; nitrates strongly absorb light in the 200–250 nm spectral region, DOC exhibits characteristic absorbance in the 250–350 nm range, and SSC dominantly affects absorption in the 350–700 nm range.^10,11^ However, several system requirements limit the practical use of UV–vis spectrophotometry to conduct water quality monitoring *in situ* in water streams. To monitor the water absorbance over a wide spectral range, UV–vis systems employ light sources, often a combination of xenon and halogen bulbs, which are inefficient, bulky, and fragile and having a limited lifetime. The requirement to spectrally resolve the broad light sources means that an extra monochromator is necessary. Lastly, Si-based photodetectors and photomultiplier tubes are the current industry standard in terms of detectors^12–14^ but are not well suited for nitrate or DOC monitoring due to their poor responsivity—typically <0.1 A/W—in the ultraviolet (UV) range. Furthermore, with a band gap energy of 1.1 eV, exposure to UV light would result in an acceleration of Si-based device aging; degradation of Si-based devices under UV exposure has been reported in UV sensors^15,16^ as well as photovoltaic modules.^17,18^ Overall, this presents a current challenge in the field to be able to monitor nitrate, DOC, and SSC levels simultaneously while ensuring that the detection device is cost-effective and compact.

The recent emergence of ultrawide-band-gap semiconductors such as gallium oxide (Ga_2_O_3_), with band gap energies nearing 5 eV (ca. 250 nm), is opening new opportunities for deep UV sensing with greater efficiency as well as reduced size and power consumption requirements compared to Si-based detectors. Although the performance of Ga_2_O_3_-based photodetectors varies widely between studies—with reported responsivities ranging from 10^–5^ to 10^5^ A/W depending on the fabrication method, crystal phase, device architecture, or measurement conditions—state-of-the-art devices now routinely achieve responsivities of 1–10^3^ A/W in the UV range, significantly greater than conventional commercial UV-enhanced photodiodes.^14,19^ Ga_2_O_3_ is typically referred to as a solar-blind photodetector, implying that the material is insensitive to light with a wavelength longer than 280 nm. However, experimental and theoretical studies have shown that a large distribution of defect states within the band gap in Ga_2_O_3_ facilitate the absorption of light at sub-band-gap wavelengths.^20,21^ In this study, we demonstrate that we can take advantage of the different electronic transition pathways (band-to-band, defect-to-band) in Ga_2_O_3_ to realize a simplified setup, allowing the monitoring of nitrate, DOC, and SSC levels in water.

## Methods

A 250 nm-thick film of undoped α-phase Ga_2_O_3_ was deposited on a *c*-plane sapphire (α-Al_2_O_3_) substrate using plasma-enhanced atomic layer deposition following the growth procedure detailed in^22^. The sample was processed into a planar photodetector device by deposition of Cr/Au metal contacts with 3/30 nm thickness in an interdigitated finger configuration with 15 μm spacing, as shown in Figure 1a,b. X-ray diffraction (XRD) diffractograms (Figure 1c) reveal a dominant reflection centered near 2θ = 40.25°, which is associated with the 0006 reflection from α-Ga_2_O_3_ and a peak at 2θ = 38.20° associated with the 111 reflection from the Au electrode (the Cr electrode is too thin to produce sufficient signal). On similarly grown samples, the rocking curve on the 0006 reflection had a full width at half-maximum (FWHM) of 22 arcsec, while the 101̅4 reflection had a FWHM of 5469 arcsec. Transmission electron microscopy (TEM) measurements supported the XRD conclusion that the material was dominantly α-Ga_2_O_3_, and Figure 1d confirms that the film consisted of α-Ga_2_O_3_ columns (lighter contrast) with inclusions of amorphous and κ-Ga_2_O_3_ between the columns (darker contrast), in line with previous findings.^22^

**Figure 1:**
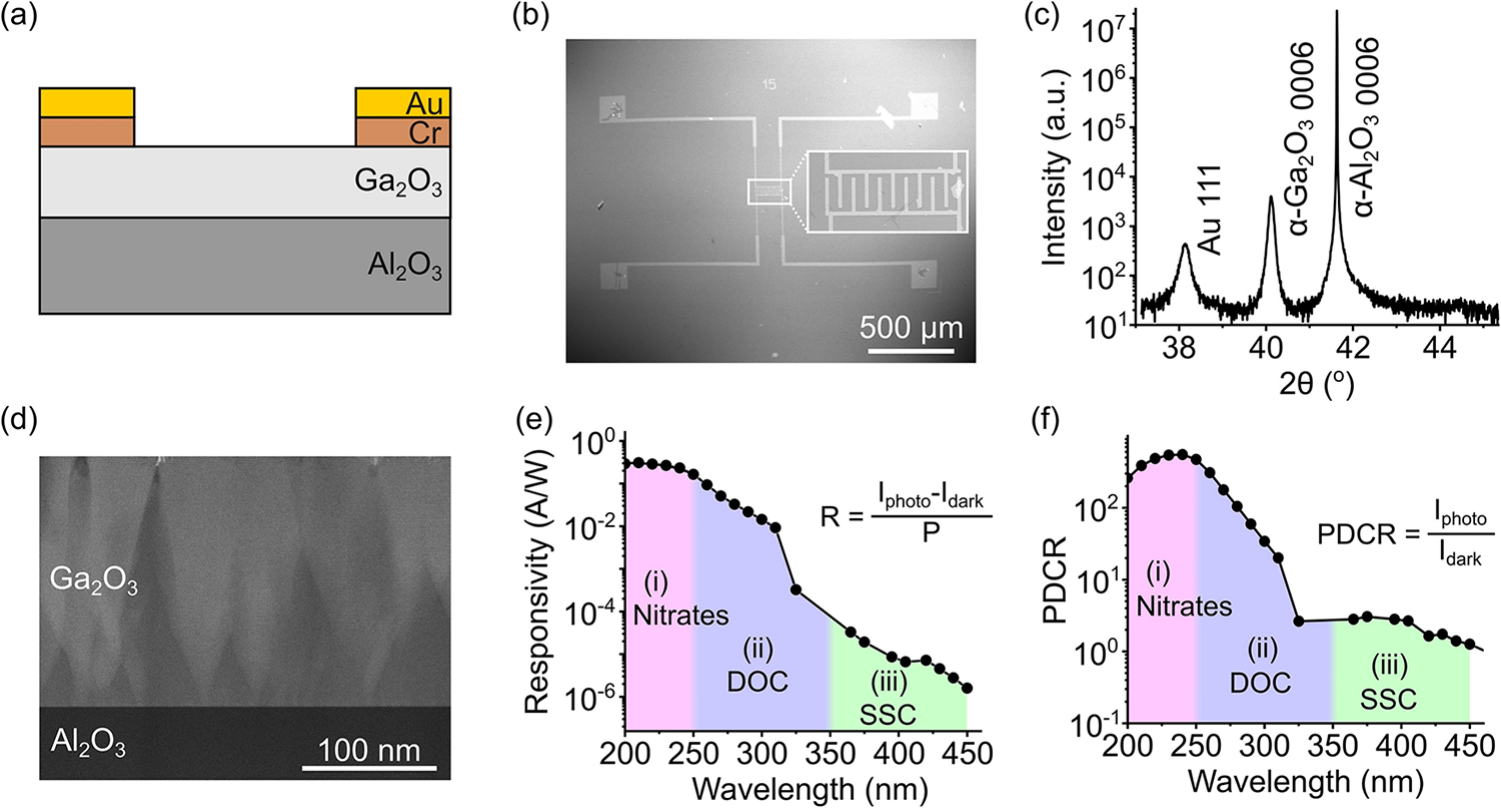
(a) Schematic of the cross-section of the Ga_2_O_3_-based photodetector. (b) Scanning electron micrograph of the device structure, with the inset showing a magnified image of the interdigitated fingers. (c) XRD diffractogram revealing reflections from the substrate (α-Al_2_O_3_), film (α-Ga_2_O_3_), and electrode (Au). (d) Cross-sectional TEM image of the device. (e) Photodetector responsivity and (f) photo-to-dark current ratio versus wavelength, with labeled characteristic absorption ranges of nitrates, DOC, and SSC based on refs 10,11 .

Optical excitation was carried out under two different regimes. To test for nitrate absorption, a Thorlabs SLS204 deuterium light source coupled to a SolarLS ML44 monochromator was used to illuminate the sample. For DOC and SSC testing, a range of Thorlabs LEDs with different peak wavelengths (250, 260, 275, 375, 405, and 465 nm) were used. The generated light was collimated through a quartz cuvette before illumination of the Ga_2_O_3_ photodetector. A Signatone probe station coupled with a Keithley 6487 picoammeter was used to apply a 10 V bias across the device and measure the photocurrent. The dark current in this device was 18 pA at 10 V bias. Upon illumination, the photocurrent was first allowed to reach a steady state with an empty cuvette, and the cuvette was subsequently filled with water containing various concentrations of nitrates, DOC, and SSC, following which the new steady-state photocurrent was recorded.

Water samples with nitrate (N-NO_3_) concentrations ranging from 1.69 to 67.7 mg/L were prepared using a 1000 mg/L NO_3_^–^ (225.8 mg/L N-NO_3_) standard for IC (Sigma-Aldrich, St. Louis, MO, USA). Water samples with DOC concentrations ranging from 1.99 to 6.7 mg/L were collected from different streams within the Attert River basin (northwest Luxembourg), acidified for preservation, and analyzed using a Torch combustion TOC analyzer (Teledyne-Tekmar, USA). Water samples with SSC ranging from 22.4 to 1247 mg/L were prepared using fine particles (ca. 30 μm).

## Results and Discussion

The key characteristics of the Ga_2_O_3_ photodetector against the illumination wavelength are displayed in Figure 1e,f. Here, the spectra are obtained through direct illumination, i.e., no light is passing through a water sample, using the spectrally filtered deuterium lamp from 200 to 300 nm and LEDs for longer wavelengths, as the deuterium lamp was too weak to generate a measurable photocurrent. The responsivity *R* describes the photogenerated current per incident optical power *P*, which provides useful insights into the fundamental properties of the photodetector. Here, we can see that the photogenerated current is maximal in the deep UV region corresponding to the band gap of the material (ca. 230–250 nm). At longer incident wavelengths, the responsivity exponentially decays from ca. 250 nm, the extent and steepness of which are indicational of disorder and defectivity in the material.^23^ The photo-to-dark current ratio (PDCR) (Figure 1f) is a related metric describing the photogenerated current *I*_photo_ compared to the dark current *I*_dark_, without normalization for incident illumination power. This metric holds great significance in technological applications because it allows for the consideration that over a broad spectral range, the available output power of illumination sources will vary significantly. The detector characteristics highlighted here demonstrate the broad response properties of Ga_2_O_3_ extending from the deep UV into the visible wavelength range. While the responsivity and PDCR plots show similar trends, we see that the decrease in the PDCR at long wavelengths is less pronounced than for the responsivity, spanning 3 orders of magnitude instead of 5, and is due to the availability of more powerful LEDs in visible and near-UV regions (typically 5–10 mW) compared to the deep UV (typically 0.5–1 mW) or spectrally resolved deuterium source (approximately 10–60 nW). Looking at the curves in more detail, we can distinguish three regions relating to the electronic band structure of the material, as schematized in Figure 2.

**Figure 2:**
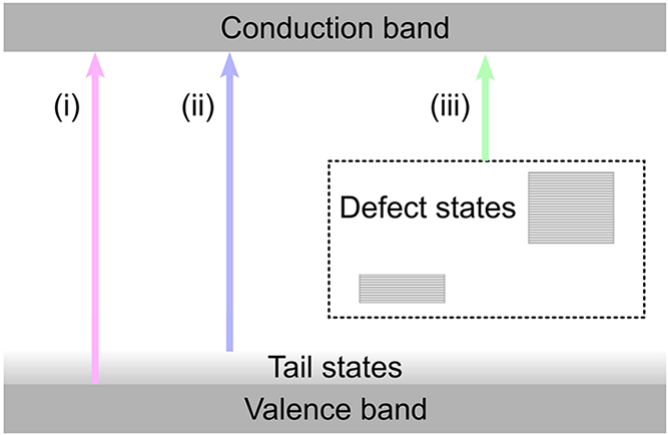
Diagram indicating the various electronic pathways to the conduction band to enable water quality monitoring for (i) nitrates, (ii) DOC, and (iii) SSC.

The first region, Region (i), extends from 200 to 250 nm. Given the reported band gap of ca. 5.1–5.3 eV for α-Ga_2_O_3_,^24–26^ the photoresponse in Region (i) is attributed to intrinsic photogeneration of free carriers in the conduction band excited from the valence band. In that region, the photocurrent is ca. 300 times greater than the dark current, demonstrating excellent sensing capabilities at these wavelengths. The UV sensing capabilities in Region (i) align perfectly with the characteristic absorption of nitrates.^10,11^ Region (ii) extends from 250 to 350 nm and is related to the photocurrent arising from carriers generated through transitions between band tail states. In a direct-band-gap semiconductor with no states in the band gap, the absorption coefficient should fall rapidly to zero at photon energies lower than the band gap energy, scaling as (*hν*–*E*_g_)^1/2^.^27^ However, as the material quality degrades, attributed to a number of factors including point defect incorporation, extended defects, and phase purity, the absorption edge broadens into the band gap, referred to as a “band tail”. Typically, for high-quality single-crystal materials, the band tail would be in the order of 10s of meV (e.g., for ZnO^28^ and GaN^29^), but is of the order of 100–200 meV for Ga_2_O_3_.^20,30^ In this study, thin-film α-Ga_2_O_3_ was used, and XRD data in Figure 1c show a single narrow peak for α-Ga_2_O_3_, but previous analysis by TEM revealed the presence of small inclusions of amorphous and κ-phase Ga_2_O_3_ (as shown in Figure 1d)^22^ as well as high densities of dislocation and grain boundaries.^31^ The results of these measurements justify the broadening of the absorption edge that we observe here. The broad exponential tail in semiconductors may be considered a negative aspect in terms of structural quality; however, it is beneficial in the context of water quality testing capabilities since a measurable photocurrent can be extracted at wavelengths below the band gap energy of the material. The photocurrent measurements and absorption characteristics shown in Figure 1e indicate that Region (ii) aligns well with the absorption characteristics of DOC.^10,11^ Finally, Region (iii) extends from 350 nm to the visible range and relates to defect-assisted transitions to the conduction band. Ga_2_O_3_ is a relatively new material, incorporating a large number of defects resulting in a high density of states distributed throughout the band gap.^21^ While the photocurrent generated through these transitions is 4–5 orders of magnitude lower than for band edge transitions, the availability of powerful LEDs (typically several mWs) in this wavelength range makes it a practical region to probe SSC contamination for the purposes of water quality testing.^10,11^ Therefore, Figure 1e,f shows that, in principle, we can take advantage of the different electronic transition pathways in α-Ga_2_O_3_ to monitor nitrate, DOC, and SSC levels in water.

We assessed the ability of the Ga_2_O_3_ photodetector to sense variations in the concentrations of these water quality parameters. According to the Beer–Lambert law,^9,32^ the luminous intensity on the photodetector (at the relevant wavelength for the water constituent) is expected to be an exponential function of its concentration; this behavior was verified for our samples using UV–vis spectrophotometry. The photodetector then converts that luminous intensity into a photocurrent following a power law (*I*_photo_ ∝ *P* ^γ^), where the coefficient γ depends on the photosensitive material as well as experimental parameters, including the illumination power itself.^23^ Where the light impinging the photodetector spans a relatively small range of power, γ can be considered constant, leading to an exponential dependence of the photocurrent with the water constituent concentration. However, for a wide power range, γ has been shown to vary for our device,^23^ leading to a nonexponential trend between the photocurrent and the water constituent concentration.

Figure 3a shows the effect of the nitrate concentration on the measured photocurrent using the above band gap 225 nm illumination. Here the excitation was generated from a deuterium light source due to the low availability of LEDs in the suitable wavelength range; however, it is noteworthy that rapid advances in UV LED technology suggest that these will soon be commercially available with optical power in the mW range,^33^ offering a significant improvement in terms of optical power, lifetime, system size, and power consumption compared to the deuterium sources. Here, we observe a nonexponential behavior which we attribute to the γ coefficient varying over the wide range of compositions tested, which leads to variations of 2 orders of magnitude in illumination power. We nevertheless observe a substantial change in the photocurrent with the nitrate concentration, which underlines the advantage of Ga_2_O_3_ over Si-based detectors owing to its greater responsivity in the deep UV range. While nonexponential, that clear trend presents a method of calibration which can be used to estimate concentration levels in untested water samples. Figure 3b shows the effect of the DOC concentration on the measured photocurrent. Using a 260 nm LED as the illumination source providing below-band-gap excitation, we observe an exponential relationship between the DOC concentration and the photocurrent with a goodness of fit R^2^ of 0.86. This demonstrates the excellent capabilities of Ga_2_O_3_ for DOC detection in water. Lastly, Figure 3c shows the impact of SSC levels on the measured photocurrent using a 465 nm LED to provide below-band-gap excitation. We observe an exponential relationship between the SSC concentration and the photocurrent with an excellent goodness of fit *R* ^2^ of 0.98. We observe a deviation from that trend for SSC levels below ca. 60 mg/L, which we ascribe to light-scattering effects induced by the initial introduction of the particles. Figure 3c illustrates the capability to advantageously use the presence of crystal defects in Ga_2_O_3_ for detecting SSC in water.

**Figure 3:**
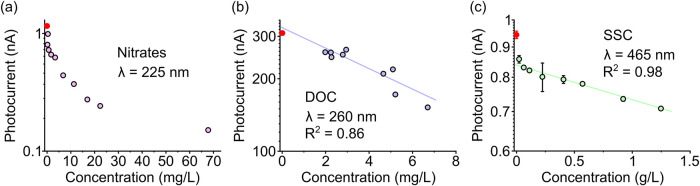
Photocurrent measurements versus the concentration of (a) nitrates using 225 nm optical excitation, (b) DOC using 260 nm optical excitation, and (c) SSC using 465 nm optical excitation. The data point marked in red signifies the photocurrent response of the cuvette filled with deionized water. Error bars are based on standard deviation from the steady-state photocurrent, which was greater for panel (c) due to the large particles in suspension.

The photocurrent measured as output of the water monitoring process is a convolution of different factors, in particular, the light source emission power, the absorption spectrum of water, the system response of the optical setup, and the spectral response of the detector. Therefore, it is expected that there will be an optimal illumination wavelength to obtain a maximal detection performance for a given water quality parameter. Figure 4 therefore illustrates the influence of the illumination wavelength on the limit of detection (3.3σ/S, with σ being the standard deviation of the response and S being the slope of the calibration curve^34^) and photocurrent contrast ((*I*_photo max_ – *I*_photo min_)/(*I*_photo max_ + *I*_photo min_)).

**Figure 4:**
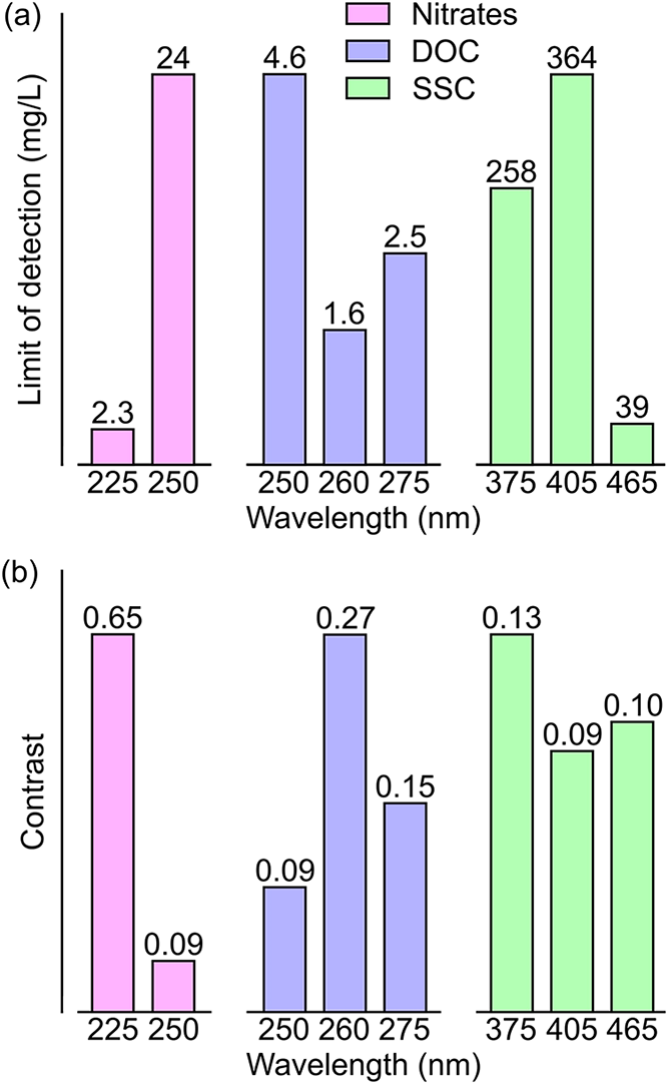
Effect of the illumination wavelength on the (a) limit of detection and (b) photocurrent contrast for water quality monitoring of nitrates (pink), DOC (purple), and SSC (green). For clarity, the *y*-axis is renormalized for each water quality parameter.

For nitrates, we show that changing the illumination wavelength from 225 to 250 nm results in a stark decrease in the limit of detection, from 2.3 to 24 mg/L, as well as a 7-fold decrease in contrast. These strong variations are most likely linked to the absorption properties of nitrates, which exhibit a significant drop in the absorption coefficient between 225 and 250 nm.^35^ Similarly, for DOC, we observe a noticeable wavelength dependence on both the limit of detection and the contrast. The 260 nm excitation yields a superior detection performance of 1.6 mg/L, compared to 250 and 275 nm illuminations, as well as a 2-fold increase in contrast. A clear dependence on the excitation wavelength is also observed for SSC contamination detection, with 465 nm excitation yielding a limit of detection of 39 mg/L, a 6- to 9-fold improvement over the limits of detection realized for 375 and 405 nm excitations. Here, we note that there is not much effect of the illumination wavelength on contrast values, which might be explained by the low response of the detector in the sub-band-gap region. In this work, excitation with wavelengths greater than 465 nm could not be used, as they resulted in a photocurrent response comparable to the dark current. Overall, these wavelength dependence results suggest the possibility to realize orthogonal determinations of water quality parameter concentrations through careful selection of the probing wavelengths. This might be achieved alongside an improvement of the detection performance through further engineering of the photosensitive material and device as well as optical design of the water monitoring setup. Importantly, this study underscores the value of defect engineering in this rapidly developing material, demonstrating that tailoring defect populations, e.g., through growth methods^36^ or post-annealing treatments,^37^ can enable new applications.

## Conclusion

In conclusion, we present α-Ga_2_O_3_ as a material showing great promise for water quality monitoring with greater sensitivity, system size, and power consumption requirements compared to Si-based technology. Ga_2_O_3_ exhibits a broad photoresponse spanning the UV and visible spectral ranges, which is a key component in detecting water constituents that absorb in different spectral regions. Making advantageous use of different carrier excitation pathways in the material, photocurrent measurements reveal three distinct regions matching the absorption characteristics of key water quality parameters. Region (i) (200–250 nm) corresponds to band-to-band transitions and is ideal for nitrate detection, Region (ii) (250–350 nm) is related to band tail transitions and fits the absorption peak of DOC, and finally, Region (iii) (350–465 nm) addresses SSC detection using defect-mediated transitions. For the selected wavelengths for water constituent testing, strong correlations were observed between concentrations of nitrates, DOC, and SSC and the photocurrent. The detection performance is also strongly dependent on the illumination wavelength, which demonstrates the good selectivity of the photodetectors. This work opens the door to more sensitive, compact, and energy-efficient systems to monitor water quality.

## Data Availability

The data that support the findings of this study are openly available from the University of Strathclyde KnowledgeBase at 10.15129/83c2259f-ad55-4340-b6d7-50b01a554394.
